# Remote evaluation of sleep to enhance understanding of early dementia due to Alzheimer’s Disease (RESTED-AD): an observational cohort study protocol

**DOI:** 10.1186/s12877-023-04288-0

**Published:** 2023-09-23

**Authors:** Jonathan Blackman, Hamish Duncan Morrison, Victoria Gabb, Bijetri Biswas, Haoxuan Li, Nicholas Turner, Amy Jolly, William Trender, Adam Hampshire, Alan Whone, Elizabeth Coulthard

**Affiliations:** 1 Bristol Medical School, University of Bristol, Bristol, BS2 8DZ UK; 2 Bristol Brain Centre, North Bristol NHS Trust, Bristol, BS10 5NB UK; 3 Faculty of Medicine, Imperial College London, London, SW7 2AZ UK; 4 Bristol Medical School, Learning & Research Building, Southmead Hospital, University of Bristol, Bristol, BS10 5NB UK

**Keywords:** Alzheimer’s disease, AD, Mild cognitive impairment, MCI, Dementia, Sleep, Circadian, Infradian, EEG

## Abstract

**Background:**

Sleep and circadian rhythm disorders are well recognised in both AD (Alzheimer’s Disease) dementia and MCI-AD (Mild Cognitive Impairment due to Alzheimer’s Disease). Such abnormalities include insomnia, excessive daytime sleepiness, decreased sleep efficiency, increased sleep fragmentation and sundowning.

Enhancing understanding of sleep abnormalities may unveil targets for intervention in sleep, a promising approach given hypotheses that sleep disorders may exacerbate AD pathological progression and represent a contributory factor toward impaired cognitive performance and worse quality of life. This may also permit early diagnosis of AD pathology, widely acknowledged as a pre-requisite for future disease-modifying therapies.

This study aims to bridge the divide between in-laboratory polysomnographic studies which allow for rich characterisation of sleep but in an unnatural setting, and naturalistic studies typically approximating sleep through use of non-EEG wearable devices. It is also designed to record sleep patterns over a 2 month duration sufficient to capture both infradian rhythm and compensatory responses following suboptimal sleep. Finally, it harnesses an extensively phenotyped population including with AD blood biomarkers.

Its principal aims are to improve characterisation of sleep and biological rhythms in individuals with AD, particularly focusing on micro-architectural measures of sleep, compensatory responses to suboptimal sleep and the relationship between sleep parameters, biological rhythms and cognitive performance.

**Methods/design:**

This observational cohort study has two arms (AD-MCI / mild AD dementia and aged-matched healthy adults). Each participant undergoes a baseline visit for collection of demographic, physiological and neuropsychological information utilising validated questionnaires. The main study period involves 7 nights of home-based multi-channel EEG sleep recording nested within an 8-week study period involving continuous wrist-worn actigraphy, sleep diaries and regular brief cognitive tests. Measurement of sleep parameters will be at home thereby obtaining a real-world, naturalistic dataset. Cognitive testing will be repeated at 6 months to stratify participants by longitudinal disease progression.

**Discussion:**

This study will generate new insights particularly in micro-architectural measures of sleep, circadian patterns and compensatory sleep responses in a population with and without AD neurodegenerative change. It aims to enhance standards of remotely based sleep research through use of a well-phenotyped population and advanced sleep measurement technology.

**Supplementary Information:**

The online version contains supplementary material available at 10.1186/s12877-023-04288-0.

## Background

AD (Alzheimer’s Disease) is the leading cause of dementia worldwide with approximately 50 million people affected [1]. Amongst this population, there is a high prevalence of sleep or circadian rhythm disturbance [2, 3] which extends to patients with Mild Cognitive Impairment due to Alzheimer’s Disease (MCI-AD) [4], illustrated by the over 60% prevalence of at least one recognised sleep disorder found in memory clinic attendees [5]. Sleep disorders and differences in chronotype are considered possible risk factors for subsequent AD dementia [6, 7], however they may also represent early manifestations of pathology [8].

Whilst aging is associated with a general deterioration in sleep quality [9], abnormalities found in individuals with AD are both more diverse [2, 10] and more severe [11]. Such disorders are a source of considerable impairment in quality of life for both patients and caregivers [12, 13].

**Fig. 1:**
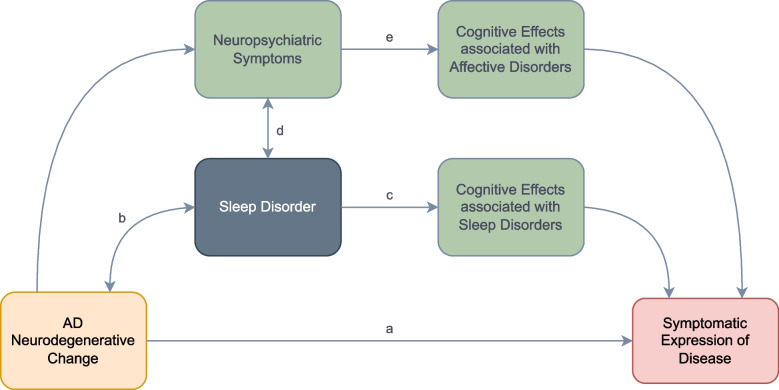
Sleep abnormalities as potential mediator of symptomatic AD. AD neurodegenerative change leading to symptomatic expression of disease through traditional pathways (**a**) but also mediated by sleep disorders through a hypothesised bidirectional relationship with AD neurodegenerative change (**b**), direct effects on cognition (**c**) and intermediary effects on neuropsychiatric symptoms (**d**) with their associated cognitive effects (**e**)

However beyond symptomatic distress, disturbances in sleep are increasingly thought to be related to clinical progression of disease (see Fig. 1).

Damage to key sleep-regulating regions is a common sequala in AD pathological change including the cholinergic basal forebrain pathways, dorsal raphe nuclei, locus coeruleus and the key pacemaker, the Suprachiasmatic Nucleus [11]. As such, sleep disturbance may arise as a direct consequence of pathological change and also behavioural change compromising sleep hygiene associated with a more severe disease state.

However, sleep disorders may also hasten the neuropathological progression of disease. Non-Random Eye Movement (NREM) sleep has been shown to play a role in clearance of beta-amyloid via the Glymphatic System [14]. Its disruption leads to alterations in cerebrospinal fluid beta amyloid in healthy adults, with rodent models demonstrating enhanced production of beta-amyloid during wake [15] and enhanced clearance during sleep [16]. Sleep may also play an important role in tau clearance [17, 18]. Further, deprivation of sleep is itself associated with poor cognitive performance [19] and increases the risk of affective disorders [20] themselves also associated with worse cognitive performance [21]. As such there are multiple pathways through which sleep disturbance may lead to an accelerated clinical presentation (see Fig. 1). Interventions to improve sleep may therefore hold promise in delaying onset or reducing the speed of progression of symptomatic AD.

Such interventions would logically be directed towards early or pre-symptomatic disease given that AD neuropathological burden is known to accumulate over decades [22]. This requires early symptomatic (or even presymptomatic) identification of AD. Given that sleep is known to be disrupted early in the course of AD [23, 24] specific abnormalities may also represent a potential biomarker of future disease – perhaps alongside emerging molecular biomarkers. Sleep measurement in AD has been subject to specific limitations that have inhibited testing theoretical relationships. For example, whilst it is specifically NREM sleep implicated as the mechanism through which clearance of AD toxic metabolites occurs, its quantification is not possible with self-reported questionnaires or wrist-based actigraphy devices and, despite this, these modalities are most often used in participants with early AD and MCI [25]. NREM measurement requires EEG analysis previously only possible through use of full in-laboratory polysomnography. Unfortunately, in-lab sleep research is compromised due to observer bias, the Hawthorne Effect (a tendency for behaviour to alter under observation) and first-night effects. Whilst clinical laboratories ensure that each participant is subject to the same conditions, there are unmeasurable person-specific environmental shifts – so in lab sleep studies never measure naturalistic sleep. Due to technological advances in sleep measurement, specifically the development of multi-channel portable EEG devices, accurate assessment of macro-architectural parameters, sleep staging and identification of micro-architectural measures of sleep in people’s homes is now possible with comparable performance to in-laboratory polysomnography (PSG) [26].

Many sleep studies are performed over relatively few nights – or even just a nap. Yet, we know there are long-term patterns in sleep i.e. infradian variability. Healthy adult sleep has been shown to vary with a periodicity of between 2 and 17 days in one study [27] and between 3 and 128 days in another which demonstrated cycles in approximately 50% of their participants [28]. This has been hypothesised to be due to either responses to process S (homeostatic factors), possibly due to inter-individual differences in the speed of build-up or dissipation of sleep need, or through variations in process C i.e. circadian processes as a response to Zeitgebers on a particular day. Variation in sleep duration has been demonstrated in Measurement of Dim-Light Melatonin Onset (DLMO) which can illuminate the nature of sleep-wake circadian entrainment [29]. Given that these processes may be impaired by Alzheimer’s pathology [11], it is important to understand whether this periodicity is altered or absent in individuals with AD-MCI, and is a factor in potential accumulation of sleep deficits. Those with AD pathology have been shown to manifest phase delay [30] but its relationship to longer-term infradian rhythms is not known. Unless we measure sleep over many nights, we cannot detect longer-term rhythmic variations in sleep-wake cycles. – and this may be a novel therapeutic target if abnormal in AD. It is also not known whether previously reported associations between evening-preference chronotype and dementia risk [7] translate to a more rapid cognitive decline.

Short study duration also precludes exploration of less common phasic events which may not arise in sufficient quantity to allow for robust analysis. For example, analysis of compensatory sleep following naturally occurring nights of poor sleep. Older adults have been shown to have a less intense and more short-lived response to prolonged wakefulness [31–33]. Orexin, as a key agent in the maintenance and regulation of sleep-wake state [34] and whose expression is affected by age [35] offers one possible explanation for this. Compromise of this axis in early AD / AD-MCI is supported by work demonstrating associations between beta-amyloid, tau and orexin and orexin receptor functionality [36–38]. This therefore raises the possibility that its compromise in early AD / MCI may drive changes in homeostatic sleep pressure thereby exaggerating loss of compensatory sleep.

AD is defined by molecular changes in brain tissues, but until recently, it has not been possible to employ a molecular, biomarker-supported diagnosis of AD ante mortem. Work instead has relied on clinical judgement and employment of established diagnostic criteria which have been demonstrated to lack sensitivity and specificity [39]. With advances in molecular technology – we can now deeply phenotype the molecular pathology of patients with cognitive disorders to understand the nature of the neuropathology. Therefore, we can for the first time start to understand the sleep deficits that align with specific pathology causing AD.

Cognitive deficits resulting from sleep deprivation and AD pathology have a huge impact on quality of life. Coupling home sleep monitoring with detailed focused cognitive testing permits evaluation of links between micro-architectural phenomena and cognition. For example, sleep spindles during slow-wave sleep have been shown to play a role in the neural plasticity required for cortical engram strengthening [40] which may help to explain positive correlations between their density and cognitive performance [41, 42]. Intensive learning tasks involving declarative memory have been linked with increased sleep spindle density in healthy adults [43]. But interestingly, increases in sleep spindle density have also been observed in rodent models following memory *retrieval* [44]. Accordingly, the very act of e.g. autobiographical events to memory could itself, theoretically, increase spindle density and mechanistically lead to increased performance on a learning task.

The RESTED-AD Programme (Remote Evaluation of Sleep To enhance understanding of Early Dementia due to Alzheimer’s Disease) was developed as a step towards addressing previous limitations and exploring new hypotheses. Its goal is to characterise sleep disturbances more deeply in well-phenotyped individuals with AD and MCI-AD utilising novel, naturalistic measurement techniques in a home environment over an extended period. Protocol design and subsequent reporting standards will follow STROBE (Strengthening the reporting of observational studies in epidemiology) guidelines [45]. We hope ultimately that insights gained may allow for targeted sleep intervention which may improve brain health in later life.

## Methods

### Hypotheses

**Fig. 2:**
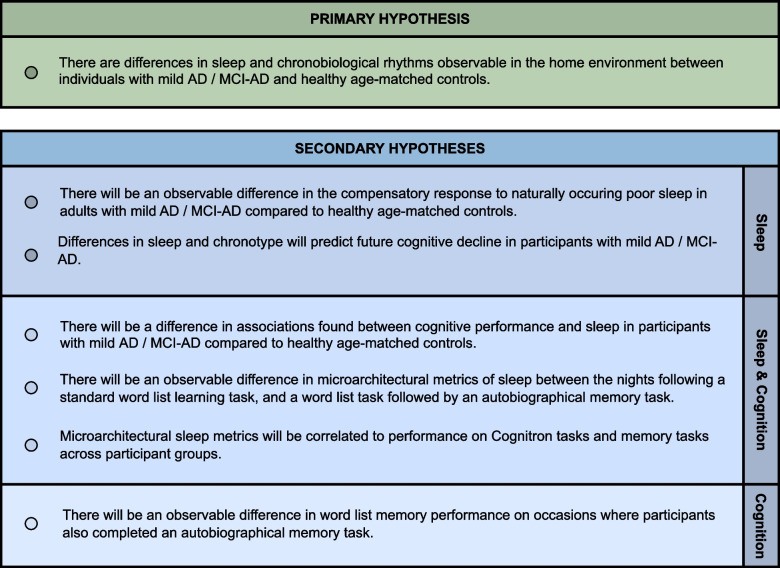
RESTED-AD hypotheses

For primary and secondary hypotheses, see Fig. 2.

### Overall study design

**Fig. 3:**
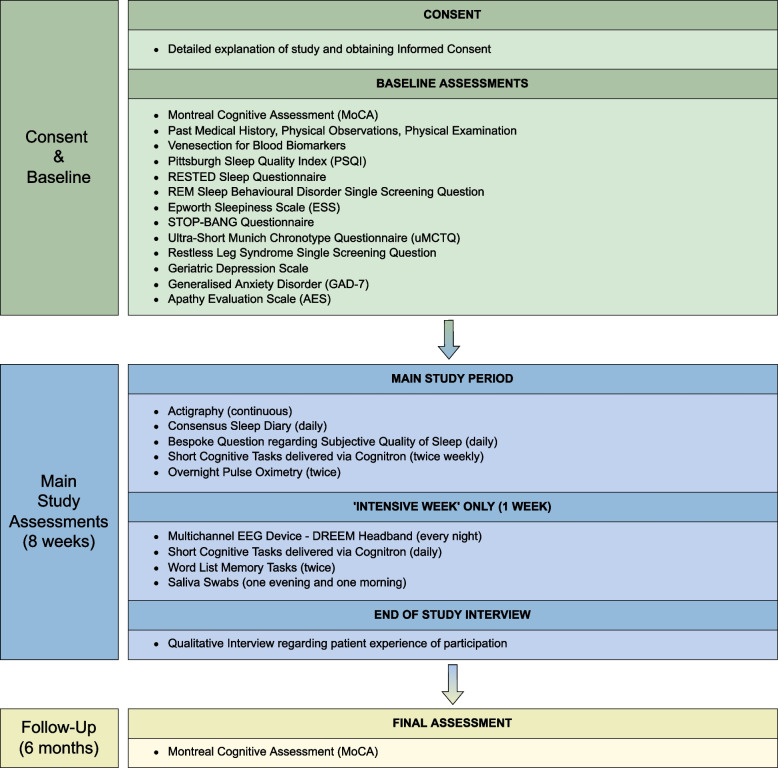
Study participant timeline

RESTED-AD is an two-arm observational cohort study, individuals with early-moderate AD / AD-MCI and healthy age-matched adults. The study received a favourable opinion from the Yorkshire & The Humber - Bradford Leeds Research Ethics Committee on 13/9/21 (ref: 21/YH/0177). Following baseline screening procedures, the main data collection phase lasting 8 weeks encompasses multiple modalities of sleep measurement combined with a series of cognitive tasks. This is followed by repeat cognitive testing at 6 months. See Fig. 3 for a summary of the overall participant timeline with further information regarding assessments provided in Data collection and measures.

### Description of participants / cohorts

Target recruitment is for 25 participants in each arm (for power calculations see Statistical analysis). The study population will be drawn from multiple sources including the North Bristol NHS Trust Cognitive Disorders Clinic, a neurology-led tertiary referral centre for investigation and treatment of memory impairment, a clinic database of previous attendees, a local charity database of healthy volunteers (BRACE-Alzheimer’s Research) and the Join Dementia Research (JDR) database – a collaboration between the National Institute for Health and Care Research (NIHR), Alzheimer Scotland, Alzheimer’s Research UK and Alzheimer’s Society designed to match healthy adults to suitable research studies. See Table 1 for full inclusion / exclusion criteria. Healthy older adults will be age-matched to participants recruited within the AD/AD-MCI arm alongside efforts to ensure approximate gender balance.

**Table 1:** Inclusion and exclusion criteria

Inclusion Criteria
Age > 50
All participants must express that they are willing to take part in this study and adhere to the study procedures
Full capacity to consent to involvement
^a^Clinical diagnosis of MCI due to AD according to standardised criteria [46] *OR*
^a^Clinical diagnosis of mild AD according to standardised criteria [47]
Exclusion Criteria
Severe medical or psychiatric co-morbidity, which, in the opinion of the investigator, may substantially impact on sleep.
Clinically significant, severe sleep disorder as defined by ICD-10 or equivalent pre-dating and / or not related to AD pathology.
Diagnosis of dementia other than AD.
Montreal Cognitive Assessment (MoCA) < 11/30

^a^For participants recruited to AD/AD-MCI arm

### Data collection

To optimise data quality and minimise observer bias, data will be collected by multiple researchers. Mandatory training will standardise the approach to tasks involving direct participant contact.

#### Demographics

Basic demographic information will be collected including age at consent, sex at birth, self-reported ethnicity, and employment status.

#### Medical screening and history

Data on medical diagnoses and medications will be retrieved from participants and electronic medical records. Participants will undergo basic physical observations. Relevant information on previous investigations relating to cognition and/or sleep (e.g., neuroimaging, CSF neurodegenerative biomarkers, overnight pulse oximetry data) will be retrieved from electronic medical records.

#### Montreal Cognitive Assessment (MoCA)

Participants will complete a MoCA with a trained researcher following consent before completing any other baseline assessments to confirm eligibility. The MoCA will be repeated at a 6-month follow-up assessment.

#### Pulse oximetry

Participants without a known Oxygen Desaturation Index (ODI) measurement within the last 6 months and who are not on CPAP treatment for diagnosed OSA will be invited to complete two nights of overnight recording using a pulse oximeter (Nonin WristOx_2_ – Model 3150).

#### Blood biomarkers

Participants will be asked to undergo a blood test for the following biomarkers of neurodegeneration: phospho-tau 181, neurofilament light chain (NFL), amyloid-beta 40 and 42, and glial fibrillary acidic protein (GFAP).

#### Sleep questionnaires

The following validated sleep questionnaires will be completed: the Pittsburgh Sleep Quality Index (PSQI) [48], Epworth Sleepiness Scale (ESS) [49], the STOP-Bang Questionnaire [50], the single screening questions for restless legs syndrome [51] and REM Sleep Behaviour Disorder [52] and the Ultra-Short Munich Chronotype Questionnaire (μMCQ) [53].

Participants will also complete the RESTED sleep questionnaire, a bespoke self-report tool capturing information pertinent to sleep health not obtained elsewhere through existing validated instruments (see SM 2 – RESTED Sleep Questionnaire).

#### Mood questionnaires

Participants will be asked to complete the Geriatric Depression Scale - Short Form (GDS-15) [54], Generalised Anxiety Disorder 7-item questionnaire (GAD-7) [55], and self-administered Apathy Evaluation Scale (AES-S) [56].

#### Mayo fluctuations composite scale

Researchers will complete the Mayo Fluctuations Composite Scale [57] for all participants.

#### Sleep diary

Participants will be asked to complete a daily sleep diary during the 8-week main study period. This will consist of the nine questions from the Consensus Sleep Diary [58] and an additional “yes/no” question written by the research team to gauge subjective sleep quality and operationalise identification of compensatory sleep: “Would you consider your sleep last night to have been much worse than normal?”. Sleep diary entries will be completed electronically using the MyDignio application, installed on a smart device. The use of Dignio software is intended to streamline the process of sleep diary data collection and allow the research team to monitor completion of the diary and related assessments.

#### Actigraphy

Wrist actigraphy will be used to evaluate rest-activity patterns during the day and macro-architectural measures of sleep and wake at night including total sleep time, sleep efficiency, night-time awakenings, and sleep latency and have been successfully used in older adult populations [59, 60]. Participants will wear a wrist actigraph (Axivity AX3, water-resistant 3-axis logging accelerometer) for the entire 8-week study period at a sampling rate of 25 Hz.

Infradian rhythms will be assessed utilising a previously described cosinor method [61]. For specific planned analyses and additional details see SM 1.

#### Short cognitive tasks

Participants will be asked to complete a set of cognitive tasks (choice reaction time, digit span, and serial object searching) at regular intervals during the study when prompted by the MyDignio application. Tasks will be scheduled on two randomly selected days per week during the main study period and daily during the intensive week. These are designed to detect fluctuations in cognitive performance which may be influenced by sleep. The tasks will be hosted on a bespoke study version of the Cognitron platform (https://cognitron.co.uk/).

#### Sleep recording with multichannel EEG device (Dreem 2) – intensive week only

Participants will be asked to wear a wireless multichannel EEG headband (Dreem 2) [26] for seven consecutive nights. Dreem 2 records, stores, and analyses EEG data collected via 5 dry sensors (two frontal sensors at F7 and F8, one ground sensor at Fp2, and two occipital sensors at O1 and O2). Sleep recording will be self-initiated by the participant. The initial recording will be discarded to prevent first night effects unless explicitly justified in future publications.

#### Word list memory tasks – Intensive week only

Participants will be shown pre-recorded videos containing 20 target words (audio and visual stimuli) in the evening. Free recall of these words will be performed immediately after the video and the following morning. To help encode these words into memory, participants will be asked to decide whether the word describes something that is ‘alive’ or ‘not alive’. The following morning, participants will be shown a second video containing target words (from the original video) and distractor (new) words and will be asked to identify the target words from the previous video.

To test whether performance of a separate task involving memory retrieval just prior to sleep may influence word list performance, the task will be completed on two occasions (with different sets of words). On one occasion, participants will be asked to complete a short autobiographical memory task just before sleep, where they are asked to recall five discrete activities/ events that occurred that day (and write them down if possible). The order in which the autobiographical task is performed will be block-randomised. Simultaneous EEG headband recordings will allow for further analysis to determine if differences are mediated by micro-architectural components of sleep (e.g. sleep spindles).

#### Salivary dim-light melatonin onset (DLMO) – intensive week only

Participants will collect seven passive drool saliva samples, one per hour, on one evening starting from 5 h before habitual bedtime and will be asked to remain awake 1 h after their usual bedtime to collect their final sample. To preserve sample integrity, participants will receive written and verbal instructions regarding hygiene and oral intake during the sampling period, guidance on maintaining dim light conditions and appropriate storage of samples until collection by the study team.

#### Salivary cortisol awakening response (CAR) – intensive week only

Participants will collect three saliva oral swabs on one morning at wake, 30 min after waking, and 60 min after waking. To preserve sample integrity, participants will receive written and verbal instructions regarding hygiene and oral intake during the sampling period, and appropriate storage of samples until collection by the study team.

### Outcome measures

**Fig. 4:**
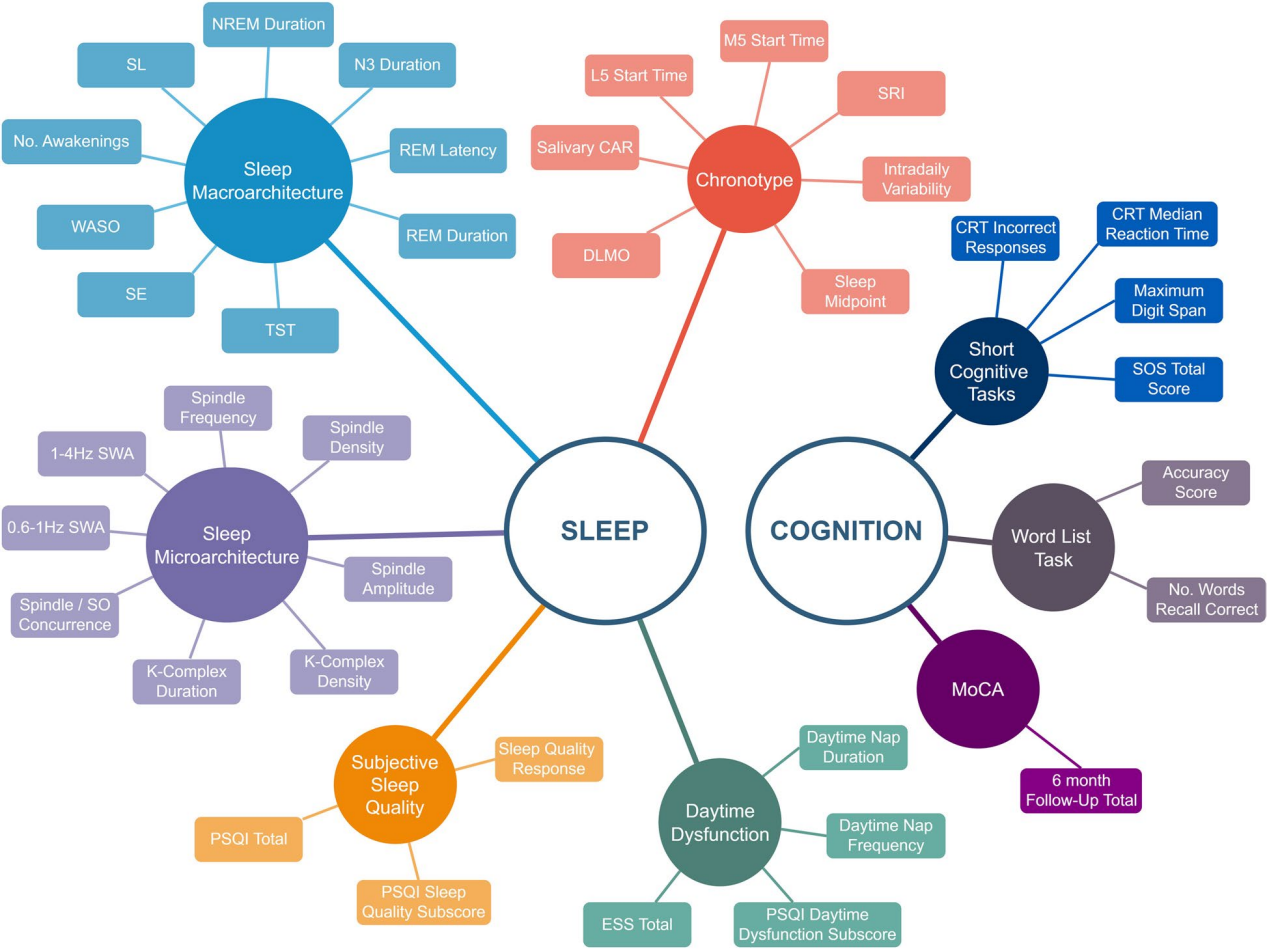
Key Outcome Measures. Abbreviations: TST – Total Sleep Time, SE – Sleep Efficiency, WASO – Wake After Sleep Onset, SL – Sleep Latency, L5 – Least Activity 5 H, M5 – Most Activity 5 H, SRI – Sleep Regularity Index, DLMO – Dim Light Melatonin Onset, PSQI – Pittsburgh Sleep Quality Index, ESS – Epworth Sleepiness Score, SWA – Slow Wave Activity, SO – Slow Oscillation, CRT – Choice Reaction Task, SOS – Self Ordered Search, MoCA – Montreal Cognitive Assessment

Figure 4 depicts recognised key sleep and cognitive outcome measures which will feed into the primary analysis plan [25].

### Statistical analysis

Data cleaning and analysis will be performed using R Studio v3.6.3 statistical software. Outlying data is anticipated as part of potential environmental / technological factors influencing cognitive performance at home and will be excluded utilising Tukey’s Criterion set at three-times the interquartile range. Sensitivity analyses will be performed to determine if participant utilisation of different electronic devices for cognitive tasks influences results. In assessing effects of the autobiographical task, intention to treat and per protocol analyses will be performed.

Comparisons will be made between both arms of the study allocated as per protocol but also across groups utilising blood biomarker status i.e. continuous levels of Aβ, tau, NFL and GFAP.

The study sample size of 50 has 80% power to detect a 7.5% difference in total sleep time and a 5% difference in sleep efficiency and based on PSG obtained normative data [62] allowing for a 10% drop out rate utilising power calculation software [63].

Basic descriptive statistics and demographics will be reported by group for relevant outcomes with appropriate tests for group comparison used depending on data normality assessed by the Shapiro-Wilkes Test.

In addressing the primary hypothesis, sleep outcome measures in Fig. 4 will be sequentially employed as dependent variables in multivariable regression models. Where the same sleep parameter is measured utilising multiple tools, a hierarchical approach will be utilised with EEG data prioritised over actigraphy data without combination of data across measurement tools. Key confounding variables will include age, sex at birth, affective symptoms (GAD-7 total score, GDS total score), medication use (binomial variables for use of each of sedative/hypnotic, antipsychotic, melatonin, ‘Over the Counter’/herbal sleep medications); suspected untreated obstructive sleep apnoea (OSA, STOP-BANG >  = 5 & not on CPAP or known OSA and declined treatment) and specific occupation / employment history (binomial variables for alarm awakening, current shift-work and historic shift-work). Regression models will be checked for adherence to standard assumptions and for multi-collinearity. Clinically plausible non-linear relationships will be assessed by use of stratification and/or quadratic terms with the best-fit model chosen for final presentation with the same approach employed for interaction terms.

There is a potential for missing data given the study complexity and duration, The approach to this will vary according to the extent and type of data loss. Reported analyses with missing data will make reference to the approach used (anticipated to be simple imputation or complete-case analysis as appropriate). Where possible, sensitivity analyses will be undertaken to examine for any impact of ‘first-night’ effects.

For approaches to identify presence and group-level differences in compensatory sleep and infradian rhythms, see SM 1 – Further Statistical Analysis Data Pertaining to Secondary Hypotheses.

## Discussion

This protocol outlines a study scoped to provide novel data concerning the relationship between sleep and AD neurodegeneration both through use of innovative sleep measurement instruments and also through enhanced duration of data collection. We hope to provide new insights into the impact of neurodegeneration on microarchitectural changes in sleep and sleep staging in a home environment. Such insights within the field lend opportunity to improve quality of life for patients but may also allow earlier identification and offer additional treatment targets for intervention.

### Supplementary Information

**Additional file 1: SM 1.** Further Statistical Analysis of Data Pertaining to Secondary Hypotheses. **SM 2.** RESTED Sleep Questionnaire.

## Data Availability

The datasets used and/or analysed during the current study are available from the corresponding author on reasonable request.
