# Supplementary material for: Remote evaluation of sleep to enhance understanding of early dementia due to Alzheimer’s Disease (RESTED-AD): an observational cohort study protocol
Source: BMC Geriatr. 2023 Sep 23;23:590. doi: 10.1186/s12877-023-04288-0 (PMC10518099; doi:10.1186/s12877-023-04288-0)

# Supplementary Materials

## SM 1 – Further Statistical Analysis of Data Pertaining to Secondary Hypotheses

### Cognitive Performance and Sleep

Performance in Cognitron tasks is anticipated to be sensitive to fatigue. Overall performance in each of the three Cognitron Tasks (Choice Reaction, Digit Span and Serial Object Searching) will be utilised as the dependent variable in separate linear regressions. For each task, separate models will incorporate one key sleep parameter anticipated to exert an effect including sleep duration, sleep efficiency and sleep fragmentation. Membership of the AD/AD-MCI group alongside confounding variables will be included as independent variables. An interaction term (AD/AD-MCI * sleep parameter [e.g. TST]) will be included to determine effects specific to the group.

### Compensatory Sleep Analysis

Compensatory sleep will be assessed utilising a random intercept lagged panel model - a structural equation modelling (SEM) approach (55). Observed longitudinal values of total sleep time (S_it_) will be decomposed into between-unit group level differences and within-unit day to day variability, which will be allowed to differ according to group membership i.e MCI/AD or healthy control participants. An overall TST mean over all participants (i) at time (t) is represented by µ_t_. Stable differences from the mean (random intercepts) between participants are represented by BS_i_. Within participant differences are represented by WS_t_ representing the difference between a participant’s observed score and the expected score (based on global mean + random intercept). Compensatory sleep represents a ‘within-person’ carry-over affect and is represented by the autoregressive parameter. A negative value of alpha implies that low TST nights are typically followed by higher TST nights. This leaves the model expressed as :-

S_it_ = µ_t_ + BS_i_ + WS_it_, where WS_it_ = αWS_it – 1_ + µ_it_

To determine if there are group level (MCI/AD vs healthy control) differences in the lagged parameter alpha, a multiple group model with no constraints across groups will be compared with a model in which alpha is constrained to be identical across groups. Comparison of these models will be made through use of the Chi-squared difference test of model fit to determine if lagged effects are dependent on group membership.

### Infradian Sleep Analysis

Initially, visual inspection will be made of TST fluctuations. Utilising previous methodology (54), a cosinor function will subsequently be fitted to the data of form :-

TST(t) = M + A × cos(2π × ω × t + φ), where t is the time (days 0-56) since study commencement, M = mean TST, A = Function Amplitude, ω Infradian Frequency (or Period^-1^), and φ = Horizontal Shift or Acrophase. A spectral density function will be calculated for each TST pattern with the largest peak frequency used for ω. Goodness of Fit will be evaluated between modelled and actual data.

## SM 2 – RESTED Sleep Questionnaire


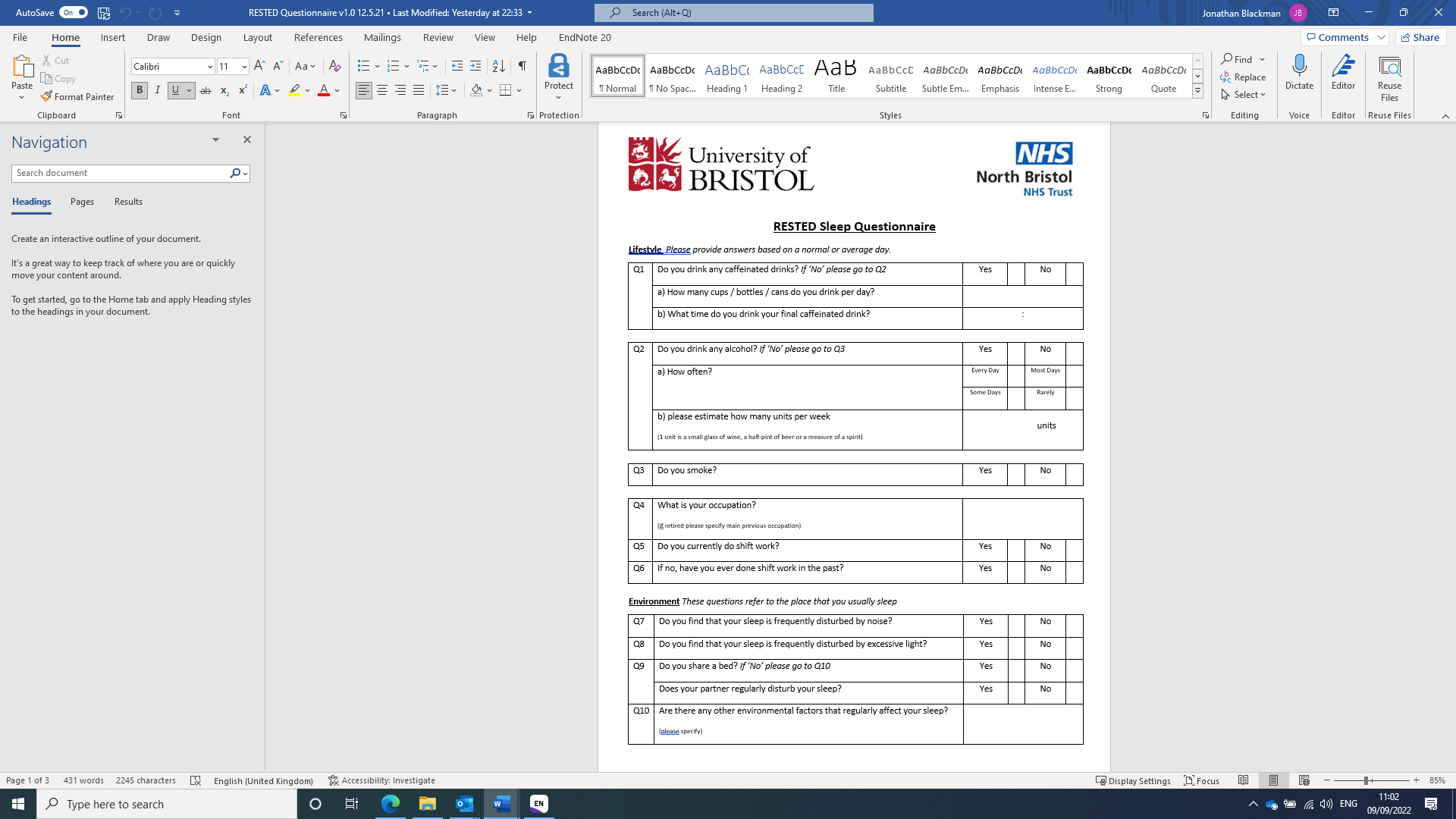


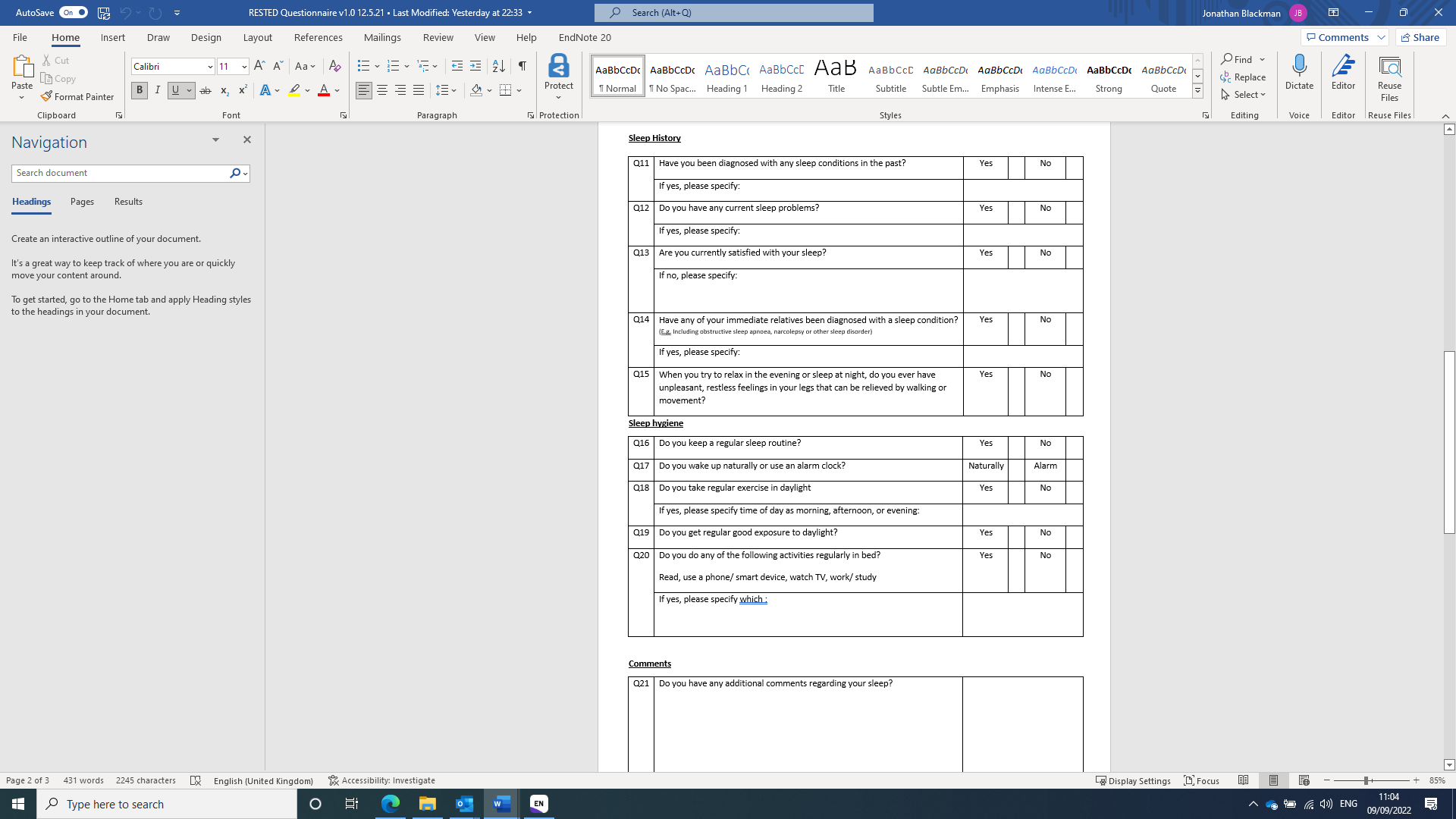

Supplement: Supplementary file 1 — Additional file 1: SM 1. Further Statistical Analysis of Data Pertaining to Secondary Hypotheses. SM 2. RESTED Sleep Questionnaire. [file 12877_2023_4288_MOESM1_ESM.docx]
